# A New 3D Tool for Assessing the Accuracy of Bimaxillary Surgery: The OrthoGnathicAnalyser

**DOI:** 10.1371/journal.pone.0149625

**Published:** 2016-02-22

**Authors:** Frank Baan, Jeroen Liebregts, Tong Xi, Ruud Schreurs, Martien de Koning, Stefaan Bergé, Thomas Maal

**Affiliations:** Department of Oral and Maxillofacial Surgery, Radboud University Nijmegen Medical Centre, Nijmegen, The Netherlands; Navoadaya Dental College and Hospital, mantralayam Road, INDIA

## Abstract

**Aim:**

The purpose of this study was to present and validate an innovative semi-automatic approach to quantify the accuracy of the surgical outcome in relation to 3D virtual orthognathic planning among patients who underwent bimaxillary surgery.

**Material and Method:**

For the validation of this new semi-automatic approach, CBCT scans of ten patients who underwent bimaxillary surgery were acquired pre-operatively. Individualized 3D virtual operation plans were made for all patients prior to surgery. During surgery, the maxillary and mandibular segments were positioned as planned by using 3D milled interocclusal wafers. Consequently, post-operative CBCT scan were acquired. The 3D rendered pre- and postoperative virtual head models were aligned by voxel-based registration upon the anterior cranial base. To calculate the discrepancies between the 3D planning and the actual surgical outcome, the 3D planned maxillary and mandibular segments were segmented and superimposed upon the postoperative maxillary and mandibular segments. The translation matrices obtained from this registration process were translated into translational and rotational discrepancies between the 3D planning and the surgical outcome, by using the newly developed tool, the OrthoGnathicAnalyser. To evaluate the reproducibility of this method, the process was performed by two independent observers multiple times.

**Results:**

Low intra-observer and inter-observer variations in measurement error (mean error < 0.25 mm) and high intraclass correlation coefficients (> 0.97) were found, supportive of the observer independent character of the OrthoGnathicAnalyser. The pitch of the maxilla and mandible showed the highest discrepancy between the 3D planning and the postoperative results, 2.72° and 2.75° respectively.

**Conclusion:**

This novel method provides a reproducible tool for the evaluation of bimaxillary surgery, making it possible to compare larger patient groups in an objective and time-efficient manner in order to optimize the current workflow in orthognathic surgery.

## Introduction

Three-dimensional (3D) treatment planning in orthognathic surgery provides surgeons with an opportunity to perform virtual osteotomies prior to surgery in order to correct dysgnathia in a predictable way and to obtain a favorable surgical outcome [1–3]. One of the key issues in obtaining a favorable postoperative outcome is an accurate transfer of the 3D planned bony movements to the patient in the operating theatre. Despite the emergence of intra-operative navigation tools, the interocclusal wafer remains the most commonly used device to transfer the 3D orthognathic planning to the patient in the operating theatre [4, 5]. The interocclusal wafer contains information concerning the positioning of maxillary and mandibular segments and guides the sagittal and transverse displacements of the maxilla and mandible during surgery. In combination with the use of a nasion pin and observing changes in dental show, vertical control of the maxilla can also be obtained intra-operatively [6, 7].

For assessing the accuracy of the postoperative outcome with regard to the 3D surgical planning, several methods have been proposed in previous studies [8, 9]. All these methods are based on the use of cephalometric landmarks to quantify differences between the virtual planning and the actual result. An inherent shortcoming of the landmark based analysis is the summation of landmark identification errors as a result of the need to identify the same landmarks multiple times. This increasing error impedes a correct interpretation of the cephalometric analysis and the actual difference between the 3D planning and the postoperative outcome.

To optimize the current way of assessing the accuracy of orthognathic surgery, this study presents a new approach to quantify the accuracy of the 3D virtual orthognathic planning, eliminating the need to identify cephalometric landmarks multiple times. The aim of this article is to validate this innovative tool, the OrthoGnathicAnalyser, in patients who underwent bimaxillary osteotomies.

## Materials and Methods

The first ten patients in 2012 with dentofacial deformities who underwent a bimaxillary surgery at the Department of Oral and Maxillofacial Surgery at the Radboud University Nijmegen Medical Centre were enrolled in this study. The inclusion criteria were a non-syndromatic dysgnathia requiring bimaxillary osteotomy and the availability of preoperative and postoperative CBCT data. Exclusion criteria were previous history of Le Fort I osteotomy or bilateral sagittal split osteotomy (BSSO), cleft palate and syndromic patients. This study was conducted in compliance with the World Medical Association Declaration of Helsinki on medical research. All patient data were anonymized and de-identified prior to analysis.

### Image acquisition

Two CBCT scans were acquired for each patient: four weeks prior to surgery and one to three weeks after surgery. Preoperative scanning was performed according to the triple scan protocol as proposed by Swennen et al. [10]. CBCT scans were acquired in the natural head position (NHP) in extended field modus (FOV: 16x22cm, scanning time 2x20s, voxel size 0,4 mm, 3D Imaging System, Imaging Sciences International Inc, Hatfield, PA, USA). Maxilim® software (Medicim NV, Mechelen, Belgium) was used to render an augmented 3D virtual head model.

### Surgery planning

The preoperative 3D augmented virtual head model was placed in the natural head position using six validated cephalometric landmarks as described by Swennen et al. [10] (Table 1). Virtual Le Fort I and BSSO osteotomies were subsequently performed on the preoperative 3D virtual head model. The maxillary and mandibular segments were moved to the desired positions in order to create 3D facial harmonization as simulated in all three dimensions by the Maxilim software (mass tensor model based soft tissue simulation). Based on the virtual planning, an interocclusal wafer was milled to transfer the virtual planning to the patient in the operating theatre.

**Table 1:** Definitions of the 3D cephalometric landmarks.

Reference landmarks	Description of landmarks	Bilateral
Nasion (N)	The midpoint of the frontonasale suture.	
Sella (S)	The center of the hypophyseal fossa.	
Porion (Por)	The most superior point of the meatus acusticus externus.	X
Orbitale (Or)	The most inferior point of the orbital rim.	X
**Landmarks maxilla**		
Upper incisor (UI)	The most mesial point of the incisor edge of the right upper central incisor.	
Mesial cusp 16	The most inferior point of mesial cusp of the crown of the right first upper molar.	
Mesial cusp 26	The most inferior point of mesial cusp of the crown of the left first upper molar.	
**Landmarks mandible**		
Lower incisor (LI)	The most mesial point of the incisor edge of the left lower central incisor.	
Mesial cusp 36	The most superior point of mesial cusp of the crown of the left first lower molar.	
Mesial cusp 46	The most superior point of mesial cusp of the crown of the right first lower molar.	
**Landmarks rami**		
Condor (Con)	The most posterior point of the mandibular ramus at the intersection with C-plane. C-plane is a plane that runs through the C-point and is parallel to the Frankfurter plane.	X
C-point (C)	The most caudal point of the sigmoid notch.	X
Gonion (Go)	The most caudal and most posterior point of the mandibular angle.	X

The Le Fort I osteotomy and BSSO were performed in general anesthesia according to Obwegeser—Dal Pont, including the Hunsuck modification. The maxilla was first positioned using the intermediate wafer and fixated with four Synthes Orthognatic 0.5 mm (DePuy Synthes Inc, West Chester, USA). Vertical control was achieved based on the intraoperative dental and gingival show. After the BSSO, the distal segment of the mandible was positioned using a second interocclusal wafer and fixed with one Champy 2.0 mm osteosynthesis plate (KLS Martin, Tuttlingen, Germany) on each side. Patients were instructed to wear tight elastics during the first week following surgery.

To evaluate the accuracy of the postoperative outcome compared to the virtual planning the following steps were carried out.

### Step 1: Registration of the postoperative 3D head model to the 3D planned model

The postoperative 3D virtual head model was rendered and registered to the preoperative 3D planned virtual head model using voxel-based matching (VBM) [11]. A subvolume that was unaffected by surgery, consisted of the anterior cranial base, zygomatic arches and forehead, was used for the registration [12].

### Step 2: Construction of a virtual triangle on each bone segment

**Fig 1:**
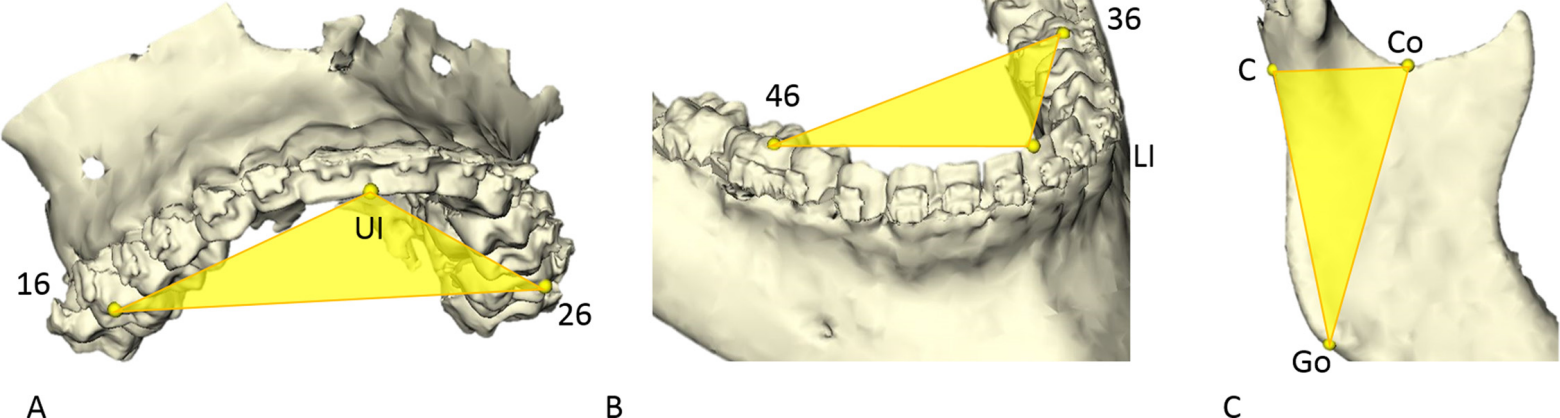
Landmarks used to create a triangle in order to obtain the 3D position and orientation. A: on the maxilla, the mesial cusp 16 (16), upper incisor (UI) and mesial cusp 26 (26). B: on the mandible, the mesial cusp 36 (36), lower incisor (LI) and mesial cusp 46 (46). C: on the proximal segment, the condor (Co), C-point (C) and gonion (Go) were identified. Virtual triangles were created based on these landmarks.

To determine the position of the maxilla, distal mandibular segment and both proximal segments, three previously validated cephalometric landmarks were placed on each bone segment (Table 1) [13–15]. The landmarks formed the vertices of a virtual triangle, which contained information on the 3D position and orientation of the bone segment (Fig 1). Triangles were constructed on the preoperative jaw segments.

### Step 3: Registration of the preoperative, 3D planned & postoperative maxillary and mandibular segments

**Fig 2:**
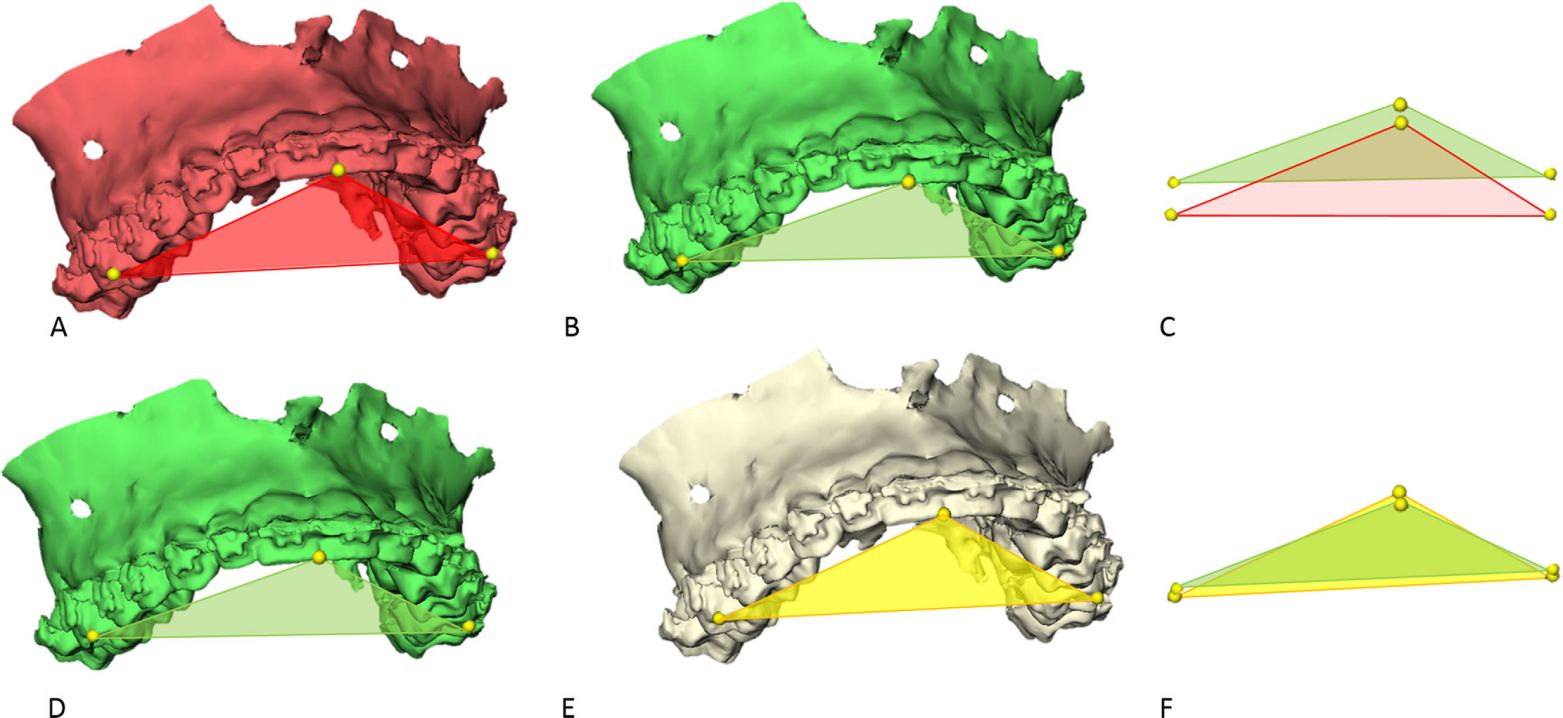
The landmarks and virtual triangle on the preoperative virtually osteotomized maxilla (A) were translated to the 3D planned position of the maxilla (B) by Maxilim. Differences between the preoperative (red) and planned (green) position of the maxilla could be seen (C). Using voxel-based registration, the 3D planned maxilla (D) was then registered to the postoperative maxilla (E). Differences between planned (green) and postoperative (yellow) position of the maxilla is displayed in (F).

The preoperative virtually osteotomized maxilla and distal mandibular segment were translated to the 3D planned position by Maxilim. The landmarks placed on the preoperative maxilla and mandible, and thus the previously constructed triangles, were translated along with the maxilla and mandible to the 3D planned position [16] (Fig 2A–2C). Consequently, the maxilla and mandibular segments were again translated from the 3D planned position to the postoperative position through voxel-based registration of the maxilla and distal segment of the mandible and surface-based registration of the proximal segments (Fig 2D–2F). In this way, the virtual triangle of each jaw segment was translated from the 3D planned position to the postoperative position.

### Step 4: Calculation of rotational and translational movements

**Fig 3:**
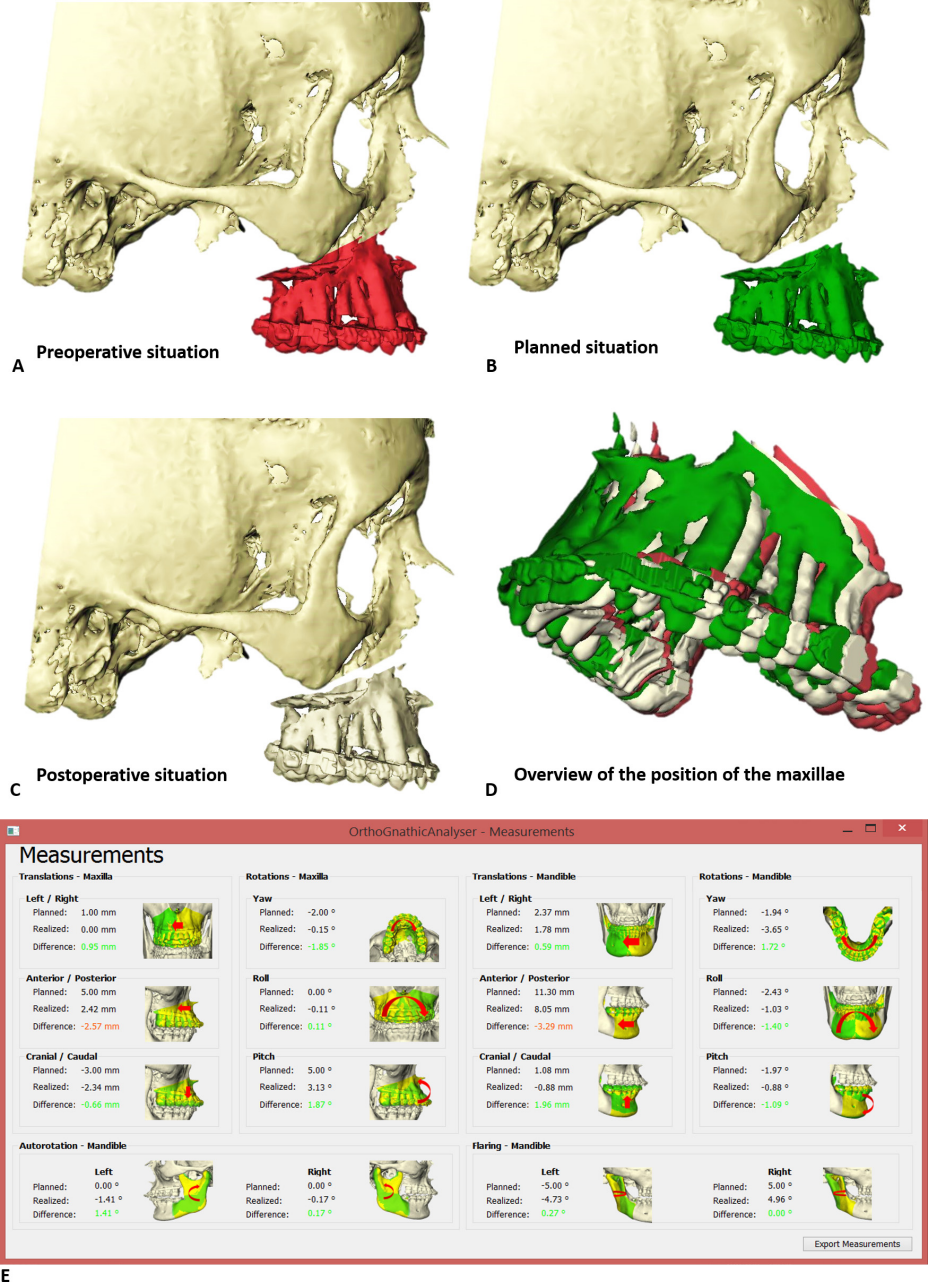
Example of a patient who underwent bimaxillary surgery. Only the maxilla is displayed. A: maxilla in the preoperative position. B: maxilla in the planned position. C: maxilla in the postoperative position. D: overview of the position of the maxillae after voxel-based registration of the head models on the anterior cranial base. E: differences between the planned surgical movement and the achieved surgical movement of the maxilla, distal and proximal mandibular segments were calculated and displayed by the OrthoGnathicAnalyser.

The coordinates of the triangles containing information on the preoperative, 3D planned and postoperative position of each jaw segment were imported into the OrthoGnathicAnalyser (Fig 3). The OrthoGnathicAnalyser was developed with C++ in Microsoft Visual Studio 2008 (Microsoft Corporation, Redmond, WA, USA) as a user-friendly interface to assess and visualize the accuracy of the translation of the 3D planning to the patient. Procrustes transformation was used [17] to match the preoperative and planned dataset towards the postoperative dataset and to calculate the translations and rotations of the virtual triangles from one dataset to another. Two transformation matrices were obtained which contained information on the translations and rotations of the maxillary and mandibular segments from preoperative position to postoperative position (surgical displacement) and from 3D planned position to postoperative position (surgical accuracy according to the 3D planning). Subsequently, the OrthoGnathicAnalyser translated these transformation matrices into clinically relevant information, such as the anterior/posterior, left/right and up/down translations as well as the pitch, roll and yaw, in a way that the discrepancies could also be visualized in a 3D viewer.

### Step 5: Clinical validation and evaluation

To validate the currently presented method and to evaluate the accuracy of the translation of 3D planning to the patients, two independent observers analyzed the CBCT data sets of ten clinical patients. Both observers performed the steps 2 to 4 independently to determine the inter-observer variability. One observer performed the steps 2 to 4 again after an interval of four weeks to assess the intra-observer variability. The mean and absolute mean differences of the surgical displacement for the maxillary, proximal and distal mandibular segments were computed. The anterior/posterior, left/right and up/down translations as well as the pitch, roll and yaw were assessed. Concerning the accuracy of the translation of 3D planning to patients, only the movements of the maxilla and distal mandibular segment were evaluated as the position of the proximal segments were not planned in 3D prior to surgery.

### Statistical analysis

Statistical data analyses were performed with IBM SPSS software, version 21.0.1 (IBM Corp., Armonk, NY, USA). Intraclass correlation coefficient (ICC) was calculated to evaluate the inter-observer and intra-observer variability for the rotational and translational measurements of the maxilla and mandible. The mean and absolute mean error in the translation of 3D planning to patients using interocclusal wafers were computed.

## Results

Six females (mean age 25,8 years, range 17–40 years) and four males (mean age 27,5 years, range 17–45 years) with skeletal Class II profile were enrolled into this study. In nine patients, an additional genioplasty was performed during the bimaxillary procedure.

### Validation of the method

The mean intra-observer and inter-observer variations in translational and rotational displacements of the maxillary and mandibular segments are displayed in Tables 2 and 3 respectively. Transverse translations were subjected to the least observer dependent errors (mean 0.037 mm) whereas vertical movements were subjected to more observer dependent variations (mean 0.24 mm). None of the mean observer dependent variation exceeded 0.25 mm. With regard to the observer dependent variations for pitch, roll and yaw, it was demonstrated that the mean intra-observer and inter-observer variations were all below 0.6°. Rotational movements of the proximal segments, however, exhibited variations up to a maximum of 1.1°. The intra-observer and inter-observer ICCs coincided with the aforementioned variations, demonstrating a very high correlation between the different measurements.

**Table 2:** The intra-observer and inter-observer variations and intraclass correlations (ICC) for measurements of the maxilla.

	Intra observer variation	95%—Cl	Inter observer variation	95%—Cl	Intra observer ICC	Inter observer ICC
**Translation AP**	0.1033 mm	(0.0440–0.1606)	0.1070 mm	(0.0691–0.1391)	0.9984	0.9983
**Translation LR**	0.0368 mm	(0.0243–0.0510)	0.0374 mm	(0.0231–0.0489)	0.9997	0.9997
**Translation UD**	0.2308 mm	(0.1110–0.3278)	0.2398 mm	(0.1602–0.2998)	0.9938	0.9934
**Pitch**	0.5995°	(0.2013–0.8923)	0.5995°	(0.4075–0.6740)	0.9717	0.9717
**Roll**	0.1430°	(0.0987–0.1818)	0.1614°	(0.0738–0.2252)	0.9960	0.9949
**Yaw**	0.0541°	(0.0366–0.0690)	0.0582°	(0.0402–0.0701)	0.9980	0.9977

AP: Anterior/Posterior, LR: Left/Right AP, UD: Up/Down. 95%-CI: 95% confidence interval.

**Table 3:** The intra-observer and inter-observer variations and intraclass correlations (ICC) for measurements of the mandibular segments.

		Intra observer variation	95%—Cl	Inter observer variation	95%—Cl	Intra observer ICC	Inter observer ICC
**Distal segment**	**Translation AP**	0.1520 mm	(0.0700–0.2235)	0.1520 mm	(0.2013–0.8923)	0.9991	0.9991
	**Translation LR**	0.0871 mm	(0.0840–0.1265)	0.1079 mm	(0.0438–0.1287)	0.9995	0.9992
	**Translation UD**	0.2136 mm	(0.1406–0.0867)	0.2136 mm	(0.0899–0.3063)	0.9930	0.9930
	**Pitch**	0.6290°	(0.3004–0.7147)	0.6327°	(0.1988–0.9731)	0.9780	0.9777
	**Roll**	0.3865°	(0.2983–0.6114)	0.4498°	(0.1993–0.5498)	0.9872	0.9827
	**Yaw**	0.0968°	(0.1006–0.0725)	0.1048°	(0.0730–0.1166)	0.9981	0.9978
**Proximal segment**	**Autorotation left**	0.7870°	(0.3428–1.2146)	0.8520°	(0.2402–1.1692)	0.9401	0.9306
	**Autorotation right**	0.9631°	(0.4444–1.6757)	1.1343°	(0.2668–1.5895)	0.9014	0.8683
	**Flair left**	0.4983°	(0.3383–1.2101)	0.8374°	(0.1401–0.7489)	0.9875	0.9654
	**Flair right**	0.3346°	(0.2263–0.9709)	0.5989°	(0.1657–0.4540)	0.9849	0.9531

AP: Anterior/Posterior, LR: Left/Right AP, UD: Up/Down. 95%-CI: 95% confidence interval.

### Accuracy of the translation of 3D planning to patients

For clinical evaluation of the accuracy of bimaxillary surgery, the postoperative result was analyzed with the OrthoGnathicAnalyser with regard to the virtual planning. The results are illustrated in Table 4 and Table 5. The left/right translation showed the lowest absolute mean difference between the 3D planning and the surgical result for both the maxilla and mandible, 0.49 mm and 0.71 mm respectively. The discrepancy between the 3D planning and the postoperative result was the greatest with regard to the vertical positioning of the maxilla and mandible, suggesting a less accurate intra-operative vertical control of the maxillary and mandibular segment using the interocclusal wafer. Furthermore, it was worth to note that in 7 out of 10 cases, the maxilla was positioned more posteriorly than in the 3D planning, with an absolute mean difference of 1.41 mm. The same tendency was found in the sagittal position of the mandible, where in 8 out of 10 cases the mandible was positioned more posteriorly than planned (absolute mean difference of 1.17 mm). The pitch of the maxilla (2.72°) and mandible (2.75°) showed the highest discrepancy between the 3D planning and postoperative result among all rotational measurements.

**Table 4:** The mean differences between the 3D planned and the postoperative position of the maxilla.

Patient	Translation AP (mm)	Translation LR (mm)	Translation UD (mm)	Pitch (degree)	Roll (degree)	Yaw (degree)
**1**	-1.44	0.04	1.19	2.60	-0.73	1.00
**2**	-0.41	0.17	-1.52	-1.67	-2.23	0
**3**	-2.63	0.11	-0.68	2.34	-1.75	3.22
**4**	0.03	-0.16	-2.85	3.82	-0.04	0.22
**5**	3.71	-0.11	-3.45	5.87	0.79	-0.68
**6**	1.05	-0.09	-2.33	3.52	1.20	-0.59
**7**	-0.38	-0.66	2.67	-4.32	0.50	2.20
**8**	-2.02	1.22	0,26	-0.77	-1.42	-0.58
**9**	-1.35	1.44	-0.64	1.04	-0.87	-0.49
**10**	-1.12	0.91	2.88	1.26	-0.82	0.75
**Mean**	-0.46	0.29	-0.45	1.37	-0.54	0.51
**Absolute mean**	1.41	0.49	1.85	2.72	1.04	0.97

Translation AP: a positive value means that the maxilla was positioned more anteriorly than planned, a negative value means that the maxilla was positioned more posteriorly than planned. Translation LR: a positive value means that the maxilla was positioned more to the right compared to the planning, a negative value means that the maxilla was positioned more to the left compared to the planning. Translation UD: a positive value means that the maxilla was displaced more cranially compared to the planning, a negative value means that the maxilla was displaced more cranially compared to the planning. Pitch: a positive value means an anti-clockwise rotation compared to the planning, a negative value means a clockwise rotation compared to the planning. Roll: a positive value means an anti-clockwise rotation around the horizontal axis compared to the planning, a negative value means a clockwise rotation around the horizontal axis compared to the planning. Yaw: a positive value means an anti-clockwise rotation around the vertical axis compared to the planning, a negative value means a clockwise rotation around the vertical axis compared to the planning.

**Table 5:** The mean differences between the 3D planned and the postoperative position of the distal mandibular segment.

Patient	Translation anterior/posterior (mm)	Translation left/right (mm)	Translation up/down (mm)	Pitch (degree)	Roll (degree)	Yaw (degree)
**1**	-1.28	0.23	1.95	3.32	0.61	-0.07
**2**	-0.71	0.35	-0.45	-1.23	0.11	-3.39
**3**	-3.03	0.44	0.20	3.51	-0.36	1.54
**4**	-0.06	-0.84	-0.08	-0.86	0.55	1.59
**5**	3.61	-0.14	-1.17	5.81	0.81	-1.00
**6**	0.36	1.17	-0.13	1.27	1.73	-1.06
**7**	-0.05	1.41	3.92	-6.71	-0.75	-0.29
**8**	-2.25	1.21	1.06	0.17	-1.31	0.76
**9**	-0.21	-0.43	1.74	2.35	-1.48	0.06
**10**	-0.10	0.25	2.50	2.30	-0.68	1.56
**Mean**	-0.37	0.25	0.96	0.99	-0.08	-0.03
**Absolute mean**	1.17	0.71	1.32	2.75	0.84	1.13

Translation AP: a positive value means that the mandible was positioned more anteriorly than planned, a negative value means that the mandible was positioned more posteriorly than planned. Translation LR: a positive value means that the mandible was positioned more to the right compared to the planning, a negative value means that the mandible was positioned more to the left compared to the planning. Translation UD: a positive value means that the mandible was displaced more cranially compared to the planning, a negative value means that the mandible was displaced more cranially compared to the planning. Pitch: a positive value means an anti-clockwise rotation compared to the planning, a negative value means a clockwise rotation compared to the planning. Roll: a positive value means an anti-clockwise rotation around the horizontal axis compared to the planning, a negative value means a clockwise rotation around the horizontal axis compared to the planning. Yaw: a positive value means an anti-clockwise rotation around the vertical axis compared to the planning, a negative value means a clockwise rotation around the vertical axis compared to the planning.

## Discussion

Three-dimensional (3D) treatment planning in orthognathic surgery provided surgeons with an opportunity to perform virtual osteotomies prior to the actual surgery in order to correct dysgnathia in a more predictable way. In order to obtain a favorable surgical outcome, an accurate translation of the 3D planning to the patients was required. To asses skeletal changes in the course of the orthognathic treatment, three distinct approaches were used in previous studies: the calculation of linear and angular differences between reference points [5, 18–23], the use of distance maps to evaluate the differences between the surface of the planned and postoperative jaw segments [24–27], and finally the computation of intra-class coefficients of reference points and reference angles [2].

In all aforementioned methods, cephalometric landmarks need to be identified multiple times on the virtual 3D model, both prior to surgery and after surgery. The error caused by the identification of landmarks ranged from 0.02 mm to 2.47 mm [15, 28, 29]. As the same landmarks had to be identified twice, the total landmark identification error could be regarded as the sum of individual landmark identification errors, which might easily exceeded the clinical relevant error margin of 0.5 mm. In relation to the error between the 3D planning and postoperative outcome which ranged from 0.03 mm to 3.71 mm in the present study (Table 4), the landmark identification error could easily have influenced a good clinical interpretation of the results. Therefore, a further reduction in the landmark identification error is crucial in the evaluation of skeletal changes throughout an orthognathic treatment.

Two approaches can be applied to overcome the landmark identification error, the fully automatic landmark recognition [30, 31] or the elimination of landmark based measurement, as proposed in this study. The essence of automatic landmark recognition is the reduction and elimination of random, observer-dependent landmark identification errors. Despite various validation studies, an identification error smaller than 2 mm is still hard to accomplish [30, 31]. A recent study by Makram et al. [32] revealed an error range of 0.3 mm to 2.8 mm for a 3D mesh based protocol for the automatic localization of cephalometric landmarks, impeding its application in the daily practice. The relatively large errors did not only arise from challenges in the computation of artificial intelligence algorithms in recognizing the anatomic relevant structures, they were also caused by streak artifacts that were frequently present as the result of orthodontic appliances, which hampered an accurate automatic recognition of anatomical structures [30].

By eliminating the necessity to identify cephalometric landmarks in each CBCT dataset through the voxel-based registration of jaw segments, as proposed in this study, the clinically relevant translational and rotational movements of each jaw segment could be computed from the rotation matrices of the jaw segments during the registration process. The translational and rotational movements of the jaw segments on the sagittal, vertical and transverse plane could be computed directly from the translation matrices by the OrthoGnathicAnalyser, instead of through interpolation from conventional cephalometric measurements. The three landmarks that were identified on each jaw segment in this study were used solely to construct the virtual triangles to allow the calculation of translation matrices, not for making cephalometric measurements. In this way, this VBM based method has eliminated the need for multiple identifications of cephalometric landmarks and is free of landmark identification errors. As a consequence, the proposed VBM method in the OrthoGnathicAnalyser overcomes measurement inaccuracies as a result of multiple landmark identification or automatic landmark recognition.

The results of the current study demonstrated an excellent reproducibility of the OrthoGnathicAnalyser in the quantification of skeletal displacements between two CBCT datasets. The very low intra-observer and inter-observer variations in measurement error of well below 0.25 mm and high ICCs (> 0.97) supported the observer-independent character of the measurements obtained from the OrthoGnathicAnalyser. The minimal variations found between the different measurements seemed to be the results of small intrinsic alignment errors caused by VBM as described by previous studies [11, 33]. As these inaccuracies were approximately half the size of a voxel (0.4 mm) and far less than the clinically accepted error margin of 0.5 mm, they can be regarded as clinically irrelevant. Compared to the measurement errors of conventional 3D cephalometry which ranged from 0.02 mm till 2.47 mm, the errors found with the OrthoGnathicAnalyser were clinically negligible. It is clear that the OrthoGnathicAnalyser can provide far more reproducible results with regard to the quantification of jaw displacements.

Despite the high consistency in the measurement of skeletal displacement by the OrthoGnathicAnalyser, it should be noted that the reproducibility of measurements concerning the proximal segments was lower than for the maxilla and distal mandibular segment. The lower intra-observer and inter-observer ICC could have been the result of the fact that the proximal segments were registered using SBM whereas the maxilla and distal mandibular segment were registered using VBM. SBM was used for the proximal mandibular segments to counteract the image artifacts as a result of the sagittal split osteotomy.

During SBM the observer had to color the area on which the SBM is performed. The input required from the observer is thus higher than in VBM, during which the observer only had to select the volume of interest. It was plausible that this more observer-dependent action in SBM could have influenced the reproducibility of the registration process of the proximal segments negatively, as described by Almukthar et al. [34] Another point of interest was the segmentation of the proximal segments. In most patients, the condyles were not completely segmented and reconstructed. The incomplete reconstruction of condyles could also have affected the accuracy of SBM, which is dependent on a good surface integrity of the matching objects.

When assessing the accuracy of the mandible, it is of major importance whether the postoperative CBCT scan was acquired in the optimal occlusion, as how the 3D planning was made. If the postoperative CBCT was acquired in a suboptimal occlusion, a discrepancy in position of the mandible between the 3D planning and the postoperative outcome could occur. To limit the role of occlusion, it is essential to perform the scanning protocol correctly.

The clinical analyses of ten patients using the OrthoGnathicAnalyser demonstrated that the interocclusal wafer provided a good control of the positioning of the maxilla and mandible on the transverse plane. In line with the findings of previous studies [35–37], the interocclusal wafer had provided far less control in the vertical direction. The vertical discrepancy between the 3D planning and the postoperative position of the maxilla was two to three times higher than in the transverse direction. Several studies suggested the intra-operative use of a nasion pin or other external reference point, to aid the positioning of the maxilla on the vertical plane [5, 38–41]. The clinical analyses of the twenty-three patients using the OrthoGnathicAnalyser showed an adequate position of the maxilla and mandible in the left/right direction with a deviation of respectively 0.32 mm and 0.75 mm which is in line with other findings [5, 42]. In the cranial/caudal direction a slightly larger deviation was congruent with other findings [7, 42]. In the maxilla the anterior/posterior deviation was ≤ 1.00 mm while in the mandible a larger deviation was seen.

With regard to the accuracy in the translation of the 3D planning to the patient in the sagittal direction, it was remarkable that all maxillae and mandiblae were positioned more posteriorly. The condylar position might be changed during surgery by muscle tone and gravity as the patient was placed in the supine position, affecting the optimal condylar seating [43]. Also the translation of the 3D planned pitch to the patient seemed to be difficult. A possible reason for the relatively large discrepancy found in the pitch can be positional errors due to bone interferences between the pterygoid plate and the osteotimized maxilla. Especially in cases in which impaction of the maxilla is planned, premature bone contact might occur. Other influential factors for the discrepancy found between the planned and the postoperative maxillary position might be the non-centric relation of the mandible when the surgical guide is used to guide the maxilla to its desired position, the use of intermaxillary fixation and a glabella pin. A clinical study with a larger population with the application of the glabella pin is now ongoing to provide more insight in the factors that may have influenced this.

The OrthoGnathicAnalyser can also be applied during the postoperative follow-up, for example to quantify the skeletal relapse one or two years postoperatively, or to compare the outcome of different surgical strategies such as maxilla versus mandible first. The additional value of new techniques such as intraoperative navigation, patient specific preoperatively fabricated splints and patient specific preoperative milled fixation plates can be evaluated systematically and objectively using this newly developed tool.

In conclusion, the OrthoGnathicAnalyser is a novel and objective tool to quantify the displacement of jaw segments in orthognathic surgery, eliminating the need for multiple landmark identification as in conventional cephalomatric analysis. With this newly developed observer independent semi-automatic tool, the accuracy of the 3D planning and surgical outcome of orthognathic surgery can be analyzed in an objective, reproducible and systematic way. With the results of the current validation study we believe that the OrthoGnathicAnalyser provides the clinicians a new powerful tool to evaluate and optimize the accuracy of 3D planning in bimaxillary surgery.

## Supporting Information

S1 Table Table with all patient data and measurements. All rotations (pitch, roll and yaw) were measurement in degrees. All translations were measured in millimeters.
(XLSX)
